# Prevalence and factors for food allergy in different populations from different regions: A protocol for a systematic review and meta-analysis

**DOI:** 10.1371/journal.pone.0261092

**Published:** 2021-12-14

**Authors:** Hua Feng, Xiujuan Xiong, Zhuo Chen, Qunying Xu, Zhongwei Zhang, Jiangao Feng, Yongning Wu

**Affiliations:** 1 State Key Laboratory of Food Science and Technology, Nanchang University, Nanchang, P.R. China; 2 School of Public Health, Nanchang University, Nanchang, P.R. China; 3 Department of Pathology, Basic Medical College of Nanchang University, Nanchang, P.R. China; 4 NHC Key Laboratory of Food Safety Risk Assessment, Chinese Academy of Medical Science Research Unit (No. 2019RU014) (China National Center for Food Safety Risk Assessment), Beijing, P.R. China; Texas A&M University College Station, UNITED STATES

## Abstract

**Background:**

To determine the prevalence of food allergy (FA) and factors associated with these occurrences in different populations from different regions.

**Materials and methods:**

The literature search will be conducted via Pubmed, Embase, Cochrane Library, Web of Science, China National Knowledge Infrastructure (CNKI), Vip and Wanfang databases. Ratio rate (RR), odds ratio (OR) and 95% confidence intervals (CIs) will be adopted to evaluate prevalence and factors for FA in different populations from different regions. When the heterogeneity is small (I^2^<50%), the fixed effect model will be analyzed, otherwise, random effects model analysis will be performed. When the heterogeneity is large (I^2^≥50%), Meta regression will be used to explore the sources of inter-study heterogeneity. When the heterogeneity is large (I^2^≥50%) and the results are statistically significant (*P*<0.05), subgroup analysis will be analyzed based on age, gender, race/region, literature quality and other factors. Funnel plots will be used to reflect reporting bias and the Begg’s test will be used to test the symmetry of the funnel plots. When publication bias occurs, “cut-and-fill” method will be adopted to adjust publication bias. And sensitivity analysis will be performed for all outcome indicators.

**Discussion:**

This meta-analysis will evaluate the prevalence of FA and factors associated with these occurrences in different populations from different regions on the basis of existing evidences. Our study may be crucial to analyze similarities and differences regarding FA between different individuals from diverse regions and eventually define preventive or diagnostic approaches specifically tailored to certain populations and regions.

**Systematic review registration:**

OSF registration number: 10.17605/OSF.IO/VQXU9

## Introduction

Food allergy (FA) is defined as an abnormal immune response to antigens introduced into the body through food, with wide spectrum of clinical manifestations, ranging from mild skin problems to severe systematic reactions [1]. The prevalence of FA is increasing on a global scale, which has become a major public health concern worldwide [2]. In the most severe case, FA can lead to anaphylaxis and might result in death within minutes [1]. FA not only affects the quality of life but also place a considerable economic burden on patients and society [3, 4]. It is of great importance to identify prevalence and factors influencing FA.

Despite the increasing prevalence of FA and its attributed public health burdens, estimating the incidence and prevalence of FA faces a huge challenge. The gold standard of FA diagnosis is the double-blind, placebo-controlled food challenge (DBPCFC) [5–8]. Nevertheless, most epidemiological studies estimated FA prevalence based on various standards, such as lay perceptions or specific immunoglobulin E (IgE) or skin prick test (SPT) sensitization to common food allergens [5, 7]. Both self-perception and allergic sensitization are known to substantially overestimate the actual prevalence of FA [9–11]. Besides, geographic region, food availability, eating habits, age, ethnicity, cultural customs, and a variety of other influencing factors make it difficult to obtain definitive prevalence data [1]. Precisely because of these complex factors, limited studies have investigated influencing factors for FA [8]. Thereby, this study will be designed to investigate the prevalence of FA and its associated factors in different populations based on diverse regions.

Herein, we plan to present a systematic and meta-analysis to provide a comprehensive report on sociodemographic, and regional patterns of FA based on different populations from different regions. We will also identify factors associated with FA in the different populations from diverse regions. This study will provide combined values and interval estimates for the prevalence of FA in different population groups and geographic regions, which may improve the prevention and control strategies of high-risk groups and high allergenic food.

### Primary objective

To determine the prevalence of FA in global context.

### Secondary objective

To identify potential influencing factors of FA.

## Methods

### Study registration

Prospective registration of this study has been approved by Open Science Framework (OSF) registries (https://osf.io/registries), and the registration number is 10.17605/OSF.IO/VQXU9. This protocol for systematic review and meta-analysis was performed according to the Preferred Reporting Items for Systematic Review and Meta-Analysis Protocols (PRISMA-P) statement.

### Ethics

This study does not require ethical approval as the study is based entirely on the published data of the ethically approved primary studies.

### Search strategy

**Fig 1:**
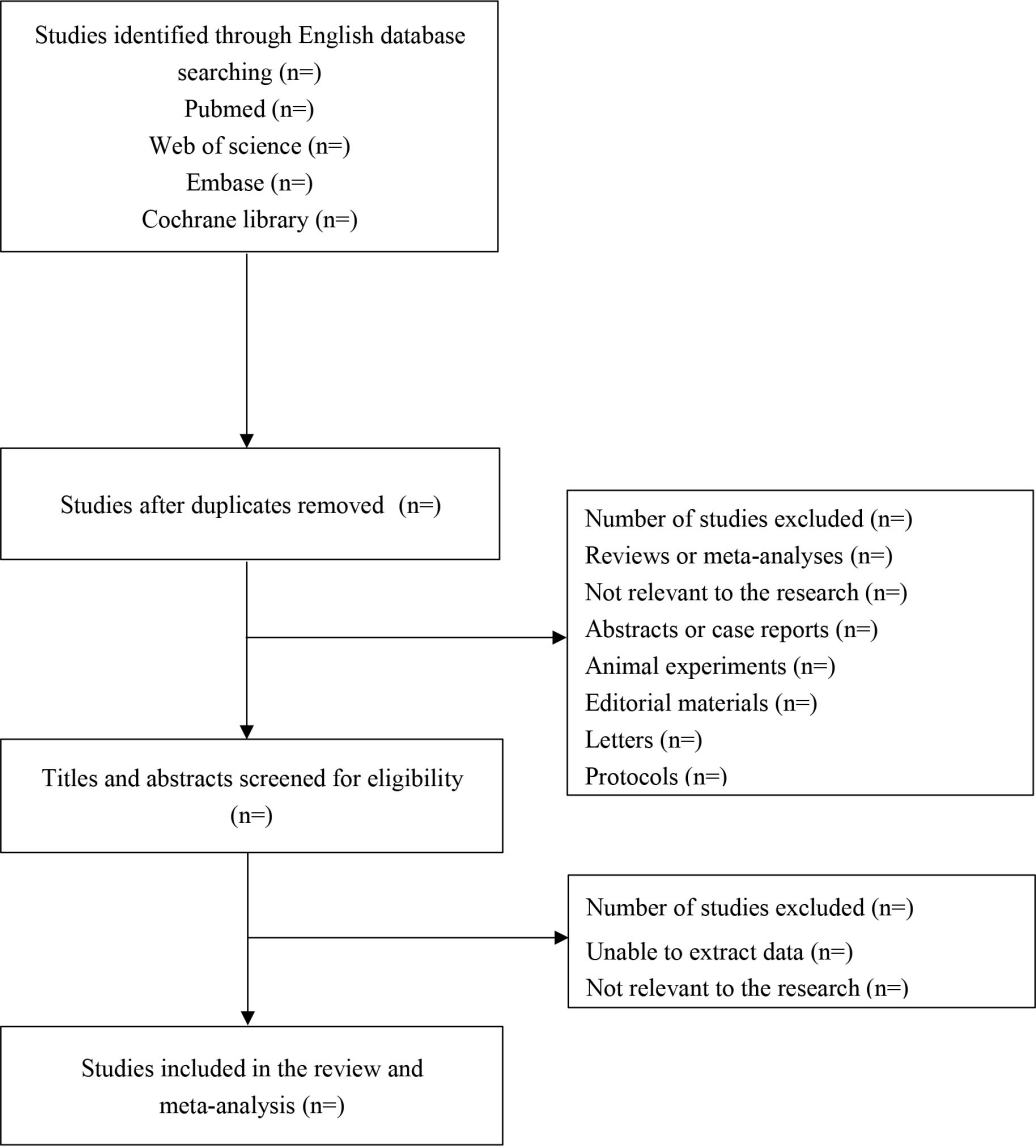
Flow diagram of search strategy and selection process.

The published literature search will be performed from inception to December 30, 2021, on PubMed (1947 to December 30, 2021), Embase (1973 to December 30, 2021), Cochrane Library (1991 to December 30, 2021), Web of Science (1985 to December 30, 2021), China National Knowledge Infrastructure (CNKI) (1999 to December 30, 2021), Vip (2000 to December 30, 2021) and Wanfang (1988 to December 30, 2021) databases. Search terms will be “Food Hypersensitivit*” OR “Hypersensitivity, Foo*” OR “Allerg*, Food” OR “Food Allerg*” AND “Prevalenc*” OR “Period Prevalenc*” OR “Prevalence, Perio” OR “Point Prevalenc*” OR “Prevalenc*, Point” AND “Factor, Ris*” OR “Risk Facto*” OR “Health Correlat*” OR “Correlat*, Health” OR “Risk Scor*” OR “Scor*, Risk” OR “Risk Factor Scor*” OR “Scor*, Risk Factor” OR “Populatio* at Risk”. The search strategy is displayed in Table 1, and the diagram showing flow of study is shown in Fig 1.

**Table 1:** PubMed search strategy.

No.	Search items
#1	(((((((Food Hypersensitivit* [Title/Abstract]) OR (Hypersensitivit*, Food [Title/Abstract])) OR (Allerg*, Food [Title/Abstract])) OR (Food Allerg* [Title/Abstract]))
#2	(((((((Prevalenc* [Title/Abstract]) OR (Period Prevalenc* [Title/Abstract])) OR (Prevalenc*, Period [Title/Abstract])) OR (Point Prevalenc* [Title/Abstract])) OR (Prevalence, Point [Title/Abstract])
#3	((((((((((((Facto*, Risk [Title/Abstract])) OR (Risk Facto* [Title/Abstract])) OR (Health Correlat* [Title/Abstract])) OR (Correlat*, Health [Title/Abstract])) OR (Risk Scor* [Title/Abstract])) OR (Scor*, Risk [Title/Abstract])) OR (Risk Factor Scor* [Title/Abstract])) OR (Scor*, Risk Factor [Title/Abstract])) OR (Populatio* at Risk [Title/Abstract]))
#4	#2 OR #3
#5	#1 AND #4

### Eligibility criteria

Inclusion criteria: (1) people with FA; (2) observational studies include cohort studies, case-control studies, and cross-sectional studies; (3) Chinese and English literature.

Excluded criteria: (1) the number of people with FA cannot be quantified; (2) animal experiments; (3) food intolerance (e.g., lactose intolerance), poisoning (food poisoning), pharmacological adverse reactions (e.g., caffeine); (4) other researches not related to the research topic; (5) case reports, editorial materials, meeting abstracts, letters, protocols, reviews, and meta-analysis.

### Methodological quality appraisal

For cohort studies and case-control studies, literature quality will be assessed using a Newcastle-Ottawa Scale (NOS) with a total score of 10, 1–4 being low quality and 5–10 being high quality. For cross-sectional studies, the quality of the literature will be assessed using the Joanna Briggs Institute (JBI) scale, which had a total score of 20 on a scale of 0–14 as low quality and 15–20 as high quality.

### Data extraction

The data will be extracted independently by two reviewers (H Feng and XJ Xiong) after the final qualified study is selected, which containing the first author’s name, year of publication, the country/region/ ethnicity, the study design, population source, sample size, age, gender, food type, number of FA events, and influencing factors of FA, etc. If information is lost, an attempt will be made to contact the original study author for relevant information. If not, the missing information will be calculated from the relevant data in the study if possible. The data extraction tables of the two authors (Z Chen and QY Xu) will be cross-checked to verify the accuracy and consistency of the extracted data. Any differences in the above process will be resolved through discussion between two authors (ZH Zhang and JG Feng), and any further differences will be arbitrated by a third author (YN Wu).

### Outcome assessment

Outcome evaluation of the literature will be from two aspects. The first one will be the prevalence of FA, that is, the proportion of people with FA in different population groups and geographic regions. The second will be factors that may affect FA, such as gender, and genetic factors (family association, Human Leukocyte Antigen (HLA), and specific genes, etc.), some potential risk factors that can be used to reduce/prevent FA, such as atopic disease manifestations (complicated with atopic dermatitis), hygiene Conditions improved, vitamin D deficiency, reduced antioxidant intake, increased use of antacids (reducing digestion of allergens), obesity (in a state of inflammation), timing and pathways of food exposure (delayed oral intake of allergens, environmental exposure in the absence of oral exposure increases the risk of sensitization and allergy) and so on.

### Statistical analysis

Stata15.1 software (Stata Corporation, College Station, TX, USA) will be used for statistical analysis. For FA prevalence, ratio rate (RR) will be adopted as the effect indicator. For factors influencing FA, the odds ratio (OR) will be used as the effect indicator for enumeration data. Each effect size will be expressed by 95% confidence intervals (CIs), and *P*<0.05 will be considered statistically significant. Heterogeneity tests will be performed for each outcome effect size. Heterogeneity statistic I will be calculated by Q test to quantify the magnitude of heterogeneity. When the heterogeneity is small (I^2^<50%), the fixed effect model will be used, otherwise, random effects model analysis will be performed.

The potential sources of heterogeneity will be investigated by subgroup and meta-regression analyses. When the heterogeneity is large (I^2^≥50%), Meta regression will be used to explore the sources of inter-study heterogeneity. When the heterogeneity is large (I^2^≥50%) and the results are statistically significant (*P*<0.05), subgroup analysis will be analyzed. Subgroup analyses will be performed by regions (Asia, America, Europe, Africa, and Oceania), populations (children, adults), diagnosis modes, etc.

Funnel plots will be used to reflect reporting bias and the Begg’s test will be used to test the symmetry of the funnel plots. When publication bias occurs, “cut-and-fill method” will be adopted to adjust publication bias.

Sensitivity analysis will be performed for all outcome indicators. In sensitivity analysis, we will analyze outcomes classified by categories of the assessed study quality variables to ascertain whether there are any relations between quality and outcomes. Low-quality studies will be removed one at a time and the meta-analysis will be performed again; we will then compare the results of the meta-analysis with and without the study being assessed. We will also conduct a cumulative meta-analysis in order of publication year to find the starting point of risk estimate becoming statistically significant and clarify the variation tendency.

## Discussion

Numerous data show that FA is very common, affecting up to 10% population, and the prevalence has increased significantly in the last two to three decades all over the world [1, 12]. An increased risk of anaphylaxis from 2009 to 2014 was noted in a survey study of government schools in Australia (>550,000 students) [13]. The US Centers for Disease Control and Prevention, using data from one question in the US National Health Interview Survey, reported that the prevalence of FA increased among children [14]. UK studies have also suggested an increase in peanut allergy [15, 16]. In addition, a cross-sectional study of infants in a single clinic in China from 1999–2009 suggested an increase in FA prevalence [17]. Nevertheless, determining the actual prevalence of FA remains elusive due to different manifestations of FA and variable severities [1]. And the prevalence of FA is also complicated by different studies proposing different definitions of allergies, evaluating specific study populations, focusing on specific foods, and using different methodologies [1]. Furthermore, geographic differences, dietary exposure, age, racial and ethnic differences, and other factors also make it difficult to obtain definitive data on the prevalence of FA [18]. Thereby, study determining the combined values and interval estimates for the prevalence of FA is significant, especially analyzing similarities and differences of different population groups and geographic regions.

Previous studies reported that family history of allergic diseases, personal history of eczema, ethnicity, and environmental factors such as pet exposure, family size were associated with the development of FA [19–23]. However, most of these factors were investigated for FA in infancy, whether these genetic and early-life environmental factors apply to different age groups and diverse populations in different regions remains a question [24]. Thus, it is necessary to determine the factors related to FA in different population groups and geographic regions to decrease the harm caused by FA.

The strengths of the current study need to be mentioned. Our study will provide (1) worldwide prevalence of FA; (2) geographical differences in prevalence of FA and related food allergens; (3) most frequent symptoms associated with specific foods; (4) risk factors associated with FA; (5) prediction of the degree of risk of the FA. Our results will potentially allow drawing conclusions about general and specific aspects of FA in various populations and diverse geographical regions. This information may be crucial to analyze similarities and differences regarding FA between different individuals from diverse regions and eventually defining preventive or diagnostic approaches specifically tailored to the certain populations and regions.

## Supporting information

S1 Checklist This is the PRISMA checklist. (DOC)
